# Anatomy Education Environment Measurement Inventory (AEEMI): a cross-validation study in Malaysian medical schools

**DOI:** 10.1186/s12909-020-02467-w

**Published:** 2021-01-14

**Authors:** Siti Nurma Hanim Hadie, Muhamad Saiful Bahri Yusoff, Wan Nor Arifin, Fazlina Kasim, Zul Izhar Mohd Ismail, Mohd Anizam Asari, Husnaida Abdul Manan @ Sulong, Asma’ Hassan, Tg Fatimah Murniwati Tg Muda, Yasrul Izad Abu Bakar, Rasheeda Mohd Zamin, Elvy Suhana Mohd Ramli, Rafidah Hod, Saiful Bahri Talip, Ku Mastura Ku Mohd Noor, Yusoff Sharizal Yusoff Azmi Merican, Muhammad Fairuz Azmi, Atikah Abdul Latiff, Madihah Rushaidhi

**Affiliations:** 1 Department of Anatomy, School of Medical Sciences, Health Campus, Universiti Sains Malaysia, Kubang Kerian, 16150 Kota Bharu, Kelantan Malaysia; 2 Department of Medical Education, School of Medical Sciences, Health Campus, Universiti Sains Malaysia, Kubang Kerian, Kota Bharu, Kelantan Malaysia; 3 Biostatistics and Research Methodology Unit, School of Medical Sciences, Health Campus, Universiti Sains Malaysia, Kubang Kerian, Kota Bharu, Kelantan Malaysia; 4 Integrative Medicine Cluster, Advanced Medical and Dental Institute, Universiti Sains Malaysia, Bertam, Kepala Batas, Penang, Malaysia; 5 Faculty of Medicine, Universiti Sultan Zainal Abidin, Medical Campus, Kuala Terengganu, Terengganu Malaysia; 6 Department of Anatomy, Faculty of Medicine, University of Malaya, Kuala Lumpur, Malaysia; 7 Anatomy Department, Faculty of Medicine, Universiti Kebangsaan Malaysia Medical Centre, Cheras, Kuala Lumpur, Malaysia; 8 Medical Education Research and Innovation Unit (MERIU), Faculty of Medicine and Health Sciences, Universiti Putra Malaysia, Seri Kembangan, Malaysia; 9 Department of Basic Medical Sciences, Faculty of Medicine and Health Sciences, Universiti Malaysia Sarawak, Kota Samarahan, Malaysia; 10 Department of Medical Science 1, Faculty of Medicine and Health Sciences, Universiti Sains Islam Malaysia, Ampang, Selangor Malaysia; 11 Department of Basic Medical Sciences, Kulliyyah of Medicine, International Islamic University Malaysia, Kuantan, Pahang Malaysia; 12 Department of Anatomy, Faculty of Medicine, Universiti Teknologi MARA, Sungai Buloh, Selangor Malaysia; 13 Anatomy Department, Faculty of Medicine, University of Cyberjaya, Cyberjaya, Selangor Malaysia; 14 Newcastle University Medicine Malaysia, Iskandar Puteri, Johor Malaysia

**Keywords:** Anatomy education environment, Learning environment, Educational climate, Validity, Reliability

## Abstract

**Background:**

The Anatomy Education Environment Measurement Inventory (AEEMI) evaluates the perception of medical students of educational climates with regard to teaching and learning anatomy. The study aimed to cross-validate the AEEMI, which was previously studied in a public medical school, and proposed a valid universal model of AEEMI across public and private medical schools in Malaysia.

**Methods:**

The initial 11-factor and 132-item AEEMI was distributed to 1930 pre-clinical and clinical year medical students from 11 medical schools in Malaysia. The study examined the construct validity of the AEEMI using exploratory and confirmatory factor analyses.

**Results:**

The best-fit model of AEEMI was achieved using 5 factors and 26 items (χ ^2^ = 3300.71 (df = 1680), *P* < 0.001, χ ^2^/df = 1.965, Root Mean Square of Error Approximation (RMSEA) = 0.018, Goodness-of-fit Index (GFI) = 0.929, Comparative Fit Index (CFI) = 0.962, Normed Fit Index (NFI) = 0.927, Tucker–Lewis Index (TLI) = 0.956) with Cronbach’s alpha values ranging from 0.621 to 0.927. Findings of the cross-validation across institutions and phases of medical training indicated that the AEEMI measures nearly the same constructs as the previously validated version with several modifications to the item placement within each factor.

**Conclusions:**

These results confirmed that variability exists within factors of the anatomy education environment among institutions. Hence, with modifications to the internal structure, the proposed model of the AEEMI can be considered universally applicable in the Malaysian context and thus can be used as one of the tools for auditing and benchmarking the anatomy curriculum.

**Supplementary Information:**

The online version contains supplementary material available at 10.1186/s12909-020-02467-w.

## Background

Educational environment is a strong predictor of student learning [1]. It comprises multifactorial elements, such as content of instruction, learning outcomes, type of curriculum, teaching methods, and strategies, learning facilities, teachers’ competencies, behavior and guidance; and peer support, that influence student motivation and ability to learn [2]. Volatility in educational systems has indirectly influenced the components of educational environments. In keeping with the development of technology, mobile learning (m-learning) and distance learning have emerged as a new generation of learning methods that require digital literacy from learners and instructors for efficient learning [3]. Nowadays, the educational environment is not only confined to spatial learning, but has extended to social learning situations, where intercultural adaptation and social equity are being emphasized to cater to globalization in learning [4, 5]. In addition, social tolerance has been identified as a contributing factor to the psychological well-being of learners, which in turn determines the success of learning in a professional and intercultural educational environment [6]. Hence, social-psychological indices have been imparted as one of the educational environment factors that should be continually monitored to ensure provision of a positive educational environment [7].

In a similar manner, anatomy education has undergone a significant evolution in various aspects of its curriculum [8, 9]. As a pillar of medical education, teaching and learning in anatomy must withstand and adapt to changes in the ecosystem of medical training [10]. Within the past two decades, the literature on anatomy education has documented various forms of technology-enhanced and educational theory-based teaching innovations to either replace or supplement traditional teaching methods (i.e., cadaveric dissections, didactic lectures, and demonstration) [11–15]. Many factors underpinned the changes in teaching methods for anatomy, which emerged since 1979 after a revamp in the medical curriculum in Malaysia [16]. For instance, the requirement for medical students to learn new medical topics in an integrated medical curriculum has resulted in a reduction of anatomy syllabus and teaching hours [17]. Nevertheless, such changes in the anatomy education system have attracted a certain degree of attention among anatomists regarding the effectivity of learning due to the increasing concern on the incompetency of anatomy knowledge and related skills among medical graduates [18, 19]. This issue has been linked to clinical errors in judgment and medicolegal litigations [20]. Notwithstanding the growing assertion of insufficient knowledge on anatomy among medical students and graduates, empirical evidence has appeared to support that such a claim is lacking [21]. Likewise, previous scholars argued that the components of the educational environment are obsolete despite robust academic discussion on changes in anatomy curricula and teaching methods [22]. In fact, debate among anatomy educators on the most effective teaching methods in anatomy and extent of teaching the subject in the medical curriculum has been long-standing [10, 21, 23]. Addressing these issues requires appropriate curriculum evaluation, whereby feedback from various stakeholders, such as medical students, should be measured to ensure empirically-based action for improvement.

With the global implementation of outcome-based education in medical training, added flexibility in teaching, and assessment methods is expected from anatomy educators, which thus requires a rapid and high adaptability to the system. An important point to be noted is that students take ownership of learning and are free to utilize any learning resources in the process [24]. Alternatively, lecturers are mere facilitators of learning, who may need to play many roles at once to ensure a smooth and efficient learning process [25]. Based on this premise, measuring students’ perception of anatomy education environment – as a feedback mechanism – is imperative for the improvement of teaching and learning of anatomy. However, to ensure accurate measurement of students’ perception of the educational environment, using a valid, and reliable tool, which is suitable within the context of anatomy education is important.

In line with such a requirement, Hadie et al. [26] developed an instrument known as the Anatomy Education Environment Measurement Inventory (AEEMI), which plays a central role in the objective of the study for several reasons. First, it helps to establish students’ perceptions of factors pertaining to educational climate that influence anatomy learning. Second, the six factors of the AEEMI, namely, students’ perception of anatomy as a subject, anatomy teachers, importance of knowledge about the subject, anatomy learning resources, self-effort in learning anatomy, and quality of histology learning facilities, are aligned with issues raised in the literature on anatomy [27–32]. Third, the AEEMI contains low-inference items of educational environment and thereby ensure accurate rating on the students’ part, which is based on experience and observation rather than opinion [33]. Several studies indicated that low-inference items in an inventory could measure users’ perceptions objectively compared with high-inference items, which capture subjective feelings and reactions [34–36]. Hence, measurement using the AEEMI will address any problems that need improvement or point out issues that are rectifiable when the objective is measurable.

The AEEMI is an instrument that measures the perception medical students regarding the educational climate specific to anatomy as a subject. Hadie et al. [26] developed the AEEMI through the Delphi technique and involved anatomists and medical educators from various countries. A validation of the inventory was conducted on pre-clinical year students in a Malaysian public medical school, where a six-factor and 25-item framework was proposed as the best-fit model for the AEEMI. The validated instrument measures the perception of students regarding anatomy as a subject, teachers, importance of knowledge on anatomy, learning resources, self-effort in learning, and quality of histology learning facilities. A five-point Likert-type scale is used to rate of agreement with the items ranging from 1 = strongly disagree to 5 = strongly agree [26]. Although the tool was demonstrated to have good content, response process, and construct validity, Hadie et al. [26] raised their concerns on the generalizability of the AEEMI items because several important items were omitted during the validation process on the basis of statistical consideration. To ensure the trustworthiness of results obtained from the measurement using the inventory, further validation is required at a global scale to take into account the variability that may exist among institutions. Hence, the study aimed to critically examine the construct validity of the AEEMI across institutions and cohorts of students.

In a broad sense, construct validity implies the accurateness of inferences made by a measurement, such that it can measure what it intends to measure [37]. Construct validity comprises five aspects, namely, content validity (i.e., items in the instrument represent the intended factor), response process validity (i.e., users of the instrument can understand the items), internal structure validity (i.e., results are replicable in a different measurement when the same inventory is used), and relationship with other variables (i.e., results correlate with those using other tools), and consequence validity (i.e., impact of the measurement) [38]. Although evidence for the validity of the AEEMI has been established in a single-center study, a cross-validation of the instrument will ensure the selection of a robust pool of items and therefore represent the global scenario of the anatomy education environment. The AEEMI will not only be valid and reliable, but also a universal inventory at least in the Malaysian context. A universal, valid, and reliable tool will ensure a successful benchmarking process of the anatomy curriculum, which in turn will enable the improvement of the curriculum. Hence, the study intended to critically evaluate the construct validity of the AEEMI across public and private medical schools in Malaysia and propose a universal framework of the AEEMI. This study aimed to answer the following research questions: (1) What is the best-fit universal model for the AEEMI? and (2) What is the internal consistency reliability of the AEEMI when administered to medical students at different phases of training across public and private medical schools in Malaysia? To answer these questions, the study hypothesized that (1) the AEEMI will demonstrate a good model fit that is universal and (2) it would show a high level of internal consistency reliability across cohorts.

## Methods

### Study design and ethical approval

A multi-center cross-sectional study was conducted at nine public and two private medical schools in Malaysia, namely, Universiti Sains Malaysia, Universiti Malaya, Universiti Kebangsaan Malaysia, Universiti Putra Malaysia, Universiti Sultan Zainal Abidin, Universiti Malaysia Sarawak, Islamic International University Malaysia, Universiti Sains Islam Malaysia, Universiti Teknologi MARA, Newcastle University Medicine Malaysia, and Cyberjaya University College of Medical Sciences. Permission and ethical clearance were obtained from the Human Research Ethics Committees (HREC), Universiti Sains Malaysia (USM/JEPeM/18040225). Prior to data collection, the institution-led researcher briefed the students from each institution on the study objective, backgrounds, methodology, and the participants’ rights and method of withdrawal. Participation in the study was on a voluntary basis, and students could withdraw from the study at any time.

### Recruitment of participants

The study recruited 1930 medical students from 11 medical schools in Malaysia across 5 years of study and phases of training. The purposive sampling method was used based on one of two criteria, namely, (1) the participant is a pre-clinical year student who is undertaking an anatomy subject under the formal medical curriculum of a participating university or (2) the participant is a clinical year student with a previous learning experience in anatomy under the formal medical curriculum of a participating university.

### Sample size

The sample size of the study was determined according to the recommendation of Costello and Osborne [39] who prescribed the best practices for factorial analysis. The authors stated that the number of subjects required in studies involving factorial analysis should be larger than five times the number of items or greater than 100 subjects. The present study decided that the minimum sample size should be 660 because the first version of the AEEMI consisted of a total of 132 items. Considering a non-response rate of 30%, the sample size was increased to 858. Hence, the minimum sample size for each institution should be 78 students.

### Research instrument

In the new cross-validated version, the researchers initially anticipated the possibility of the inclusion of items omitted by Hadie et al. [26]. Hence, the current study used the first version of the AEEMI (Additional file 1), which contains 11 factors and 132 items. This version achieved a positive scale-level content validity index/average (S-CVI/Ave) of more than 0.80 for eight factors and borderline S-CVI/Ave ranging from 0.77 to 0.79 for the three remaining factors [26]. The 132-item version of the inventory required students to rate the items using a five-point Likert-type scale (1 = strongly disagree, 2 = disagree, 3 = not sure, 4 = agree, and 5 = strongly agree).

### Data collection process

The guided self-administered questionnaire was distributed during face-to-face sessions in lecture halls or classes by respective institution-led researchers. The time estimated for the completion of the questionnaire was 15 min. Completion of the AEEMI was voluntary, and the students were informed that their progress in the medical course will remain unaffected should they decline participation.

### Data analysis

Exploratory and confirmatory factor analyses were used to evaluate the psychometric properties of the AEEMI. Exploratory factor analysis (EFA) was performed at the outset of data analysis to determine the factor loading for each item and to explore extractable factors using Statistical Package for the Social Sciences (SPSS) version 26 (IBM Corp., Armonk, NY). The correlation matrix of the items was considered factorable when the Kaiser–Meyer–Olkin (KMO) value exceeded 0.5, and Bartlett’s test was significant [40]. The principal axis factoring method was applied to extract factors, out of which factors with eigenvalues above 1 were retained. Varimax rotation was applied to optimize the factor loading of each item on the extracted factors. Items with factor loadings of more than ±0.4 were selected for confirmatory factor analysis (CFA) [41].

CFA was performed using Analysis of Moment Structure version 24 (SPSS Inc., Chicago, IL) [42]. Goodness-of-fit indices were determined to assess the model fit of the AEEMI models, which were considered fit when all indices achieved the minimum requirement, as shown in Table 1. Contributions of the observed variables (i.e., AEEMI items) to the latent variables (i.e., AEEMI factors) were estimated by standardized factor loadings, whereby a high factor loading indicates a high contribution of the item to the factor [48]. In addition, the relationship between changes in parameter constraints and reduction of chi square values is reflected by modification indices (MIs) [48]. The study used the MI value as an indicator for selecting any observed variables fit for retention in the framework [48]. However, removal of the observed variables was based on the opinion of a content expert and literature review [49].

**Table 1:** Goodness-of-fit indices used to signify model fit

Name of category	Name of index	Level of acceptance
Absolute fit^a^	Root Mean Square of Error Approximation (RMSEA)	< 0.08 [43]
	Goodness-of-fit Index (GFI)	> 0.9 [44]
Incremental fit^b^	Comparative Fit Index (CFI)	> 0.9 [44]
	Tucker–Lewis Index (TLI)	> 0.9 [45]
	Normed Fit Index (NFI)	> 0.9 [46]
Parsimonious fit^c^	Chi Square/Degree of Freedom (Chisq/df)	< 5 [47])

^a^Absolute fit: Measures overall goodness-of-fit for the structural and measurement models collectively. This type of measure does not make any comparison to a specified null model (incremental fit measure) or adjust for the number of parameters in the estimated model (parsimonious fit measure)

^b^Incremental fit: Measures goodness-of-fit that compares the current model to a specified “null” (independent) model to determine the degree of improvement over the null model

^c^Parsimonious fit: Measures goodness-of-fit representing the degree of model fit per estimated coefficient. This measure attempts to correct for any “overfitting” of the model and evaluates the parsimony of the model compared to the goodness-of-fit

In addition to factorial analyses, internal consistency reliability was investigated to assess the internal structure of the AEEMI, which was determined by reliability analysis using SPSS version 26 (IBM Corp., Armonk, NY). Cronbach’s alpha coefficient reflected the results, where values higher than 0.7 were considered to be of high internal consistency, whereas those between 0.6 and 0.7 were considered to be of satisfactory internal consistency [50].

## Results

### Demographic profile of the participants

Out of the 1930 respondents, 51.6% were pre-clinical year students who were actively involved in formal anatomy classes, whereas the remaining 48.4% were clinical year students who learned clinical applied anatomy integrated into other clinical subjects. Table 2 summarizes the demographic distribution of the participants.

**Table 2:** Demographic distributions of participants

Variable	n	%
Institutions		
Public medical school		
Universiti Malaya	323	16.7
Universiti Sains Malaysia	219	11.3
Universiti Kebangsaan Malaysia	44	2.3
Universiti Malaysia Sarawak	140	7.3
Universiti Sultan Zainal Abidin	253	13.1
Universiti Putra Malaysia	225	11.7
Islamic International University Malaysia	132	6.8
Universiti Teknologi MARA	384	19.9
Private medical school		
Cyberjaya University College of Medical Science	91	4.7
Newcastle University Medicine Malaysia	119	6.2
Year of study		
1st	368	19.1
2nd	628	32.5
3rd	328	17.0
4th	470	24.4
5th	136	7.0
Phase of training		
Pre-clinical	996	51.6
Clinical	934	48.4

### Factorial analyses

CFA was used to confirm the dimensionality of the AEEMI, where it is established that the AEEMI measures multiple factors of the anatomy education environment. CFA on the original 6-factor and 25-item model (Model A) by Hadie et al. [26] indicated poor model fit as it reached a normed fit index (NFI) value of less than 0.9. To improve the model fit of the original 6-factor AEEMI model, the study performed stepwise item removal based on the MIs, standardized residual covariances, and standardized factor loadings, which resulted in a second model with 20 items (Model B).

Considering the removal of a large number of items, many important items could have been removed. Thus, the present study intended to identify an alternative model by EFA followed by CFA. Result of the EFA revealed that the correlation matrix of the items was factorable with a KMO value of 0.7 and a significant Bartlett’s test of sphericity. Items with factor loadings of than ±0.4 were omitted.

Two models that load on five factors were proposed on the basis of CFA, namely, a five-factor model with 20 items and a five-factor model with 26 items. Both models were found fit as satisfactory goodness-of-fit indices were achieved for the models. Table 3 summarizes the goodness-of-fit indices for the original six-factor and 25-item version (Model A), modified six-factor and 20-item version (Model B), new five-factor and 20-item version (Model C), and new five-factor and 26-item version (Model D).

**Table 3:** Proposed models of AEEMI and goodness-of-fit indices

Model	χ ^2^ statistics (df)	*P*-value	Goodness-of-fit indices					
			χ ^2^/df	RMSEA	GFI	CFI	NFI	TLI
**Model A:** Six-domain model (25 items) ^a^	4208.91 (1536)	< 0.001	2.740	0.023	0.905	0.920	0.880	0.906
**Model B:** Six-domain model (20 items) ^b^	1782.66 (906)	< 0.001	1.968	0.017	0.949	0.968	0.935	0.958
**Model C:** Five-domain model (20 items) ^c^	2036.36 (960)	< 0.001	2.121	0.018	0.943	0.960	0.928	0.953
**Model D:** Five-domain model (26 items) ^d^	3300.71 (1680)	< 0.001	1.965	0.017	0.929	0.962	0.927	0.956

^a^Based on the 25-item AEEMI by Hadie et al. (2017)

^b^Revised AEEMI with 20 items based on Hadie et al. (2017)

^c^The new 20-item AEEMI proposed by this study

^d^The final 26-item AEEMI based on the combination of Models 2 and 3

*χ* ^*2*^*/df* Chi square/degree of freedom, *RMSEA* root mean square of error approximation, *GFI* goodness-of-fit index, *CFI* comparative fit index, *NFI* normed fit index, *TLI* Tucker–Lewis index

### Internal consistency

Reliability analysis was performed on the three models with good model fit (i.e., Models B, C, and D). Cronbach’s alpha values for each construct of the AEEMI for Model B ranged from 0.369 to 0.901, which indicated poor to high reliability, respectively. In the same context, Models C and D ranged from 0.621 to 0.927, which indicated acceptable to high reliability. Tables 4, 5, and 6 present Cronbach’s alpha and standardized factor loading values for the factors in Models B, C, and D, respectively.

**Table 4:** Standardized factor loading and Cronbach’s alpha for Model B (six-factor and 20-item version)

Factor	Item	SFL	Cronbach’s alpha
Students’ perceptions of anatomy teachers	Q56. Teachers are well prepared.	0.86	0.901
	Q58. Teachers are enthusiastic to teach.	0.79	
	Q55. Teachers are knowledgeable.	0.82	
	Q54. Teachers are approachable.	0.79	
	Q63. Teachers are good role model for learning anatomy.	0.70	
Students’ perceptions of the importance of anatomy knowledge	Q115. I can apply my anatomical knowledge in clinical years.	0.58	0.802
	Q114. My anatomy knowledge helps me to understand other medical subjects.	0.52	
	Q46. The anatomy topics prepare me for clinical years.	0.77	
	Q48. The anatomy topics are relevant to future profession.	0.75	
	Q47. Relevant anatomy topics are reemphasized in clinical years.	0.66	
Students’ perception of anatomy subject	Q100. Learning anatomy is fun.	0.82	0.723
	Q99. Anatomy is an interesting subject.	0.85	
	Q109. I am confident to answer anatomy questions well.	0.42	
Students’ perceptions of anatomy learning resources	Q87. Prosected specimens are accessible.	0.45	0.554
	Q71. Learning facilities are well maintained.	0.61	
	Q76. Practical sessions are well organized,	0.57	
Students’ perception of their efforts to learn anatomy	Q21. I use anatomy models/specimens to learn anatomy.	0.39	0.369
	Q37. Anatomy examinations help me to identify my weaknesses about anatomy knowledge.	0.59	
Students’ perceptions of the quality of histology learning facilities	Q93. The quality of the microscopes provided for studying histology slides is poor.	0.62	0.704
	Q95. Poor quality of histology slides.	0.87	

*SFL* standardized factor loading; overall Cronbach’s alpha value = 0.817

**Table 5:** Standardized factor loading and Cronbach’s alpha for Model C (five-factor and 20-item version)

Factor	Item	SFL	Cronbach’s alpha
Students’ perceptions of the importance of anatomy knowledge	Q19. Learning anatomy prepared me to be a good doctor.	0.61	0.749
	Q46. The anatomy topics prepare me for clinical years.	0.74	
	Q48. The anatomy topics are relevant to future profession.	0.74	
	Q114. My anatomy knowledge helps me to understand other medical subjects.	0.54	
Students’ positive perceptions of anatomy teachers	Q57. Teachers know how to make session interesting.	0.77	0.880
	Q59. Teachers inspire me to learn more.	0.87	
	Q60. Teachers speak clearly.	0.77	
	Q63. Teachers are good role model for learning anatomy.	0.82	
Students’ negative perceptions of anatomy teachers	Q65. Teachers get irritated when asked questions.	0.73	0.856
	Q66. Teachers scold for mistakes.	0.81	
	Q67. Teachers avoid eye contact.	0.84	
	Q78. The teachers criticize students when they make errors.	0.62	
Students’ perception of anatomy subject	Q31. I am confident to answer most of the anatomy questions.	0.61	0.830
	Q107. I can explain difficult anatomy concepts to my friends.	0.67	
	Q108. I am confident to teach anatomy to others.	0.86	
	Q109. I am confident to answer anatomy questions well.	0.85	
Students’ perceptions of anatomy learning resources	Q71. Learning facilities are well maintained.	0.41	0.648
	Q86. Prosected specimens are adequate.	0.67	
	Q87. Prosected specimens are accessible.	0.74	
	Q91. Anatomy plastic models are adequate in number.	0.46	

*SFL*standardized factor loading; overall Cronbach’s alpha value = 0.745

**Table 6:** Standardized factor loading and Cronbach’s alpha for Model D (five-factor and 26-item version)

Factor	Item	SFL	Cronbach’s alpha
Students’ perceptions of anatomy knowledge relevance	Q19. Learning anatomy prepared me to be a good doctor.	0.60	0.786
	Q46. The anatomy topics prepare me for clinical years.	0.76	
	Q47. Relevant anatomy topics are reemphasized in clinical years.	0.65	
	Q48. The anatomy topics are relevant to future profession.	0.75	
	Q114. My anatomy knowledge helps me to understand other medical subjects.	0.53	
	Q115. I can apply my anatomical knowledge in clinical years.	0.59	
Students’ positive perceptions of anatomy teachers	Q54. Teachers are approachable.	0.69	0.927
	Q55. Teachers are knowledgeable.	0.72	
	Q56. Teachers are well prepared.	0.75	
	Q57. Teachers know how to make sessions interesting.	0.77	
	Q58. Teachers are enthusiastic to teach.	0.85	
	Q59. Teachers inspire me to learn more.	0.85	
	Q60. Teachers speak clearly.	0.77	
	Q63. Teachers are good role models for learning anatomy.	0.79	
Students’ negative perceptions of anatomy teachers	Q65. Teachers get irritated when asked questions.	0.86	0.856
	Q66. Teachers scold for mistakes.	0.79	
	Q67. Teachers avoid eye contact.	0.85	
	Q78. The teachers criticize students when they make errors.	0.59	
Students’ perception of anatomy subject mastery	Q31. I am confident to answer most of the anatomy questions.	0.64	0.830
	Q107. I can explain difficult anatomy concepts to my friends.	0.62	
	Q108. I am confident to teach anatomy to others.	0.84	
	Q109. I am confident to answer anatomy questions well.	0.87	
Students’ perceptions of anatomy learning resources	Q71. Learning facilities are well maintained.	0.57	0.621
	Q76. Practical sessions are well organized.	0.59	
	Q87. Prosected specimens are accessible.	0.50	
	Q91. Anatomy plastic models are adequate in number.	0.49	

*SFL* standardized factor loading; overall Cronbach’s alpha value = 0.820

### The final model

**Fig. 1:**
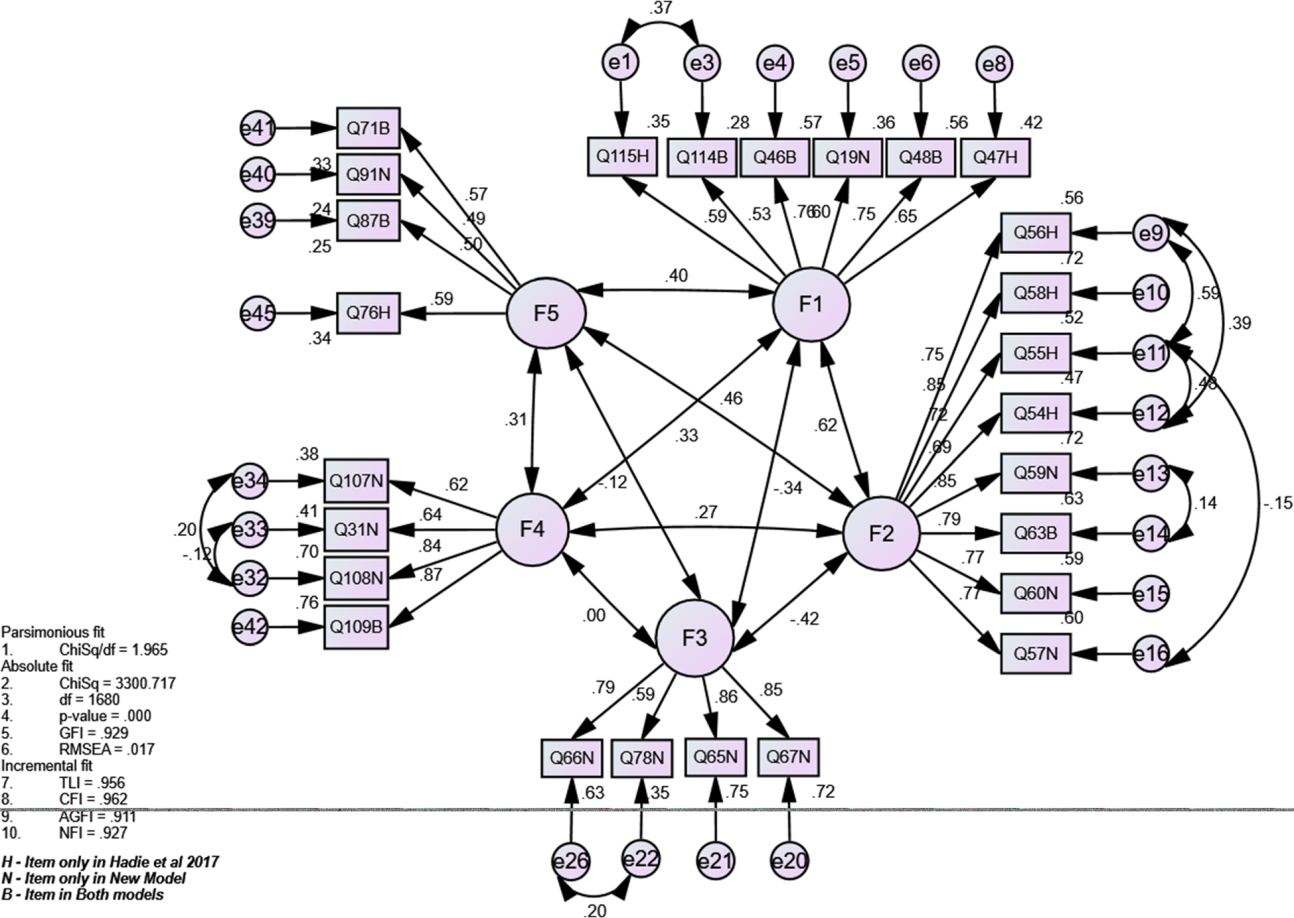
Standardized factor loading of the domains in the final model of Anatomy Education Environment Measurement Inventory

Considering the results, Model D, which contains five factors and 26 items, was selected as the final model (AEEMI-26). Analysis revealed that the model has achieved model fit with high goodness-of-fit indices. The reliability of each factor in the model ranged between satisfactory and high reliability. In addition, correlation values between factors were less than 0.85 (absolute value), which indicated good discriminant validity [48], as shown in Fig. 1.

## Discussion

The study contributes several important pieces of evidence to support the validity of the AEEMI. First, the best-fit model of the AEEMI (AEEMI-26) consists of five factors with a total of 26 items. The factors are related to anatomy knowledge relevance, positive, and negative aspects of anatomy teachers, mastery of the anatomy subject, and anatomy learning resources. Second, out of 26 items, 25 obtained standardized factor loadings of approximately 0.5, which indicate that the AEEMI-26 possesses a positive factorial structure that supports internal structure validity. Third, the five factors were independent and exclusive from one another as the correlation values between factors were less than 0.85, thus signifying the discriminant validity of AEEMI-26. Fourth, the internal consistency of the five factors ranged from satisfactory to high with Cronbach’s alpha values between 0.62 and 0.92. Fifth, the AEEMI-26 showed an overall high internal consistency and internal structure across medical schools and years of study, thus confirming that the AEEMI-26 is a cross-valid and reliable tool for measuring the anatomy education environment. Lastly, results suggested that the AEEMI-26 is a promising benchmarking tool for measuring the quality of anatomy education environment in medical schools, especially in the Malaysian context.

The AEEMI-26 measures the quality of the anatomy education environment based on the students’ point of view in terms of anatomy knowledge relevance, anatomy teachers, anatomy subject mastery, and anatomy learning resources. These factors are defined according to the items in the AEEMI-26 that represent them. For instance, anatomy knowledge relevance refers to the usability, applicability, and transferability of anatomy knowledge in future clinical practice either as medical students or practitioners. Anatomy teacher refers to the behaviors, skills, and enthusiasm, which may be negative or positive. Anatomy subject mastery reflects the ability of medical students to answer anatomy questions and explain anatomy contents to others with confidence and clarity. Anatomy learning resources refer to learning tools and materials used to support medical students for learning anatomy. In comparison to the initial version of the AEEMI that comprised six factors and 25 items (AEEMI-25) [26], the factors in the AEEMI-26 are more robust and comprehensive because they show better representation of the anatomy education environment covering common areas across different medical schools and phases of training. Furthermore, the six factors of the AEEMI-25 are covered by the AEEMI-26, for example, the effort to learn anatomy, which is a factor in the AEEMI-25, is covered under the anatomy subject mastery in the AEEMI-26; and the quality of histology learning facilities in the AEEMI-25 is part of anatomy learning resources in the AEEMI-26.

The AEEMI-26 measures the ability of students to grasp the subject (anatomy subject mastery), connection of a subject with real practice (anatomy subject relevance), teaching behaviors (anatomy teachers), and supports for learning (anatomy learning resources). Based on this notion, the AEEMI-26 measures the aspects of the educational environment that are in line with several educational environment frameworks [51]. In addition, the model agrees with the view of [52], who suggested that “The environment of the medical is notable, not only because it derives from and is a manifestation of the curriculum, but because the environment is a determinant, of the behavior of the medical school’s students and teachers.” These facts support the strength of the AEEMI-26 in measuring important aspects of the anatomy education environment at various medical school settings. Notably, although much has been discussed about the educational environment in medical and allied health sciences education [51, 52], less effort has been exerted to explore the educational climate in a specific medical environment, such as anatomy. Despite the reduction of the number of factors, the constructive alignment between the AEEMI-26 factors and global issues of anatomy education indicates that the proposed model covers the relevant constructs of the anatomy education environment and thus enhances its validity credential [26].

Approximately 96% of the items achieved standardized factor loadings of approximately 0.5, which indicates that the AEEMI-26 has a good factorial structure that supports its internal structure validity [40]. A high standardized factor loading indicates the high degree of contribution of an item to the expression of the concept represented by a factor. In contrast, the AEEMI-25 [26] achieved standardized factor loadings of at least 0.5 for approximately 84% of the items, which suggests that the AEEMI-26 with five factors has a better conceptual representation. Internal structure validity is an important indicator that supports the validity of a measurement [38–42, 48–50]. Thus, establishing the internal structure of the AEEMI-26 across medical schools in Malaysia is essential to support its cross-validity in measuring the anatomy education environment.

The AEEMI-26 achieved good discriminant validity with correlations of less than 0.85 between factors [48]. This finding indicates that its five factors are independent and exclusively measure the anatomy education environment. Discriminant validity is established when factors achieve low correlation to one another [53]. The good discriminant validity of the AEEMI-26 can be attributed to the robust and rigorous development of a refined version of the AEEMI, thus comprising well-defined and non-redundant factors with a good pool of items [26]. Moreover, this finding has a significant impact on its psychometric credential as a valid and generalizable instrument for measuring the anatomy education environment, as data were derived from 11 medical schools in Malaysia. The study proposes that the AEEMI-26 should be further validated in other countries to provide additional evidence to support its credential as a global measurement of the anatomy education environment.

In addition, the study provided evidence that the AEEMI-26 is a reliable instrument. Reliability is broadly defined as the ability of a measurement tool to produce consistent results over time and with repetition, which is commonly expressed as internal consistency and stability [54]. The five factors of the AEEMI-26 showed satisfactory to high levels of reliability as Cronbach’s alpha values ranged from 0.62 to 0.92. In comparison, the factors of [26] AEEMI-25 reached Cronbach’s alpha values ranging from 0.60 to 0.8, which indicated a nearly similar level of reliability with AEEMI-26. Compared to a more established instrument – i.e., the Dundee Ready Educational Environment Measurement –Cronbach’s alpha of the factors of the AEEMI-25 ranged from 0.58 to 0.82 in a sample of Malaysian medical students [54], which suggests a reliability comparable to other educational environment scales. Moreover, the present study provided essential evidence to support the internal consistency of AEEMI-26 across 11 medical schools in Malaysia, thus strengthening its validity for measuring the anatomy education environment.

Hence, the AEEMI-26 displayed a valid internal structure as evidenced by its independent factorial structure and high levels of reliability across medical schools and training phases. The finding suggests that it is a valid and reliable inter-institutional tool for measuring the anatomy education environment, which has several implications for the area of anatomy curriculum improvement. The use of concise 26-item validated AEEMI could minimize instances of rating errors, and therefore provide a more reliable feedback to educators on what should be improved to cater for the students learning needs. In many instances, improvement of education system was documented as a result of high-quality management system that included feedback as one of its measurement tools as emphasized by Hattie and Timperley, [55].

Hence, it is postulated that AEEMI would be able to provide significant information of the current anatomy curriculum that needs to be improved; and henceforth address the incompetency of anatomy knowledge and related skills among medical graduates. These facts are important evidence for the proposal of AEEMI-26 as a promising benchmarking tool for measuring the quality of the anatomy education environment in medical schools, particularly in the Malaysian context. Furthermore, it can be used as a global benchmarking tool to identify the strengths and areas for improvement, facilitate the formulation of an institutional development plan (IDP) to build on strengths and fill identified gaps, prioritize IDP interventions, and monitor progress and achievements [56].

### Limitations

Despite favorable outcomes that support the validity of the AEEMI-26, the study has several limitations that should be considered for future research and interpretation. Although the AEEMI initially comprised 132 items, it underwent extensive removal of items, that is, 106 items were removed during the validation process. Many items that may reflect anatomy education environment in other countries (i.e., cadaveric dissection and learning using anatomy software) are not listed under AEEMI-26. The remaining items reflect the real practice in Malaysian medical schools, whereby cadaveric dissection is not widely practiced because of shortage of cadavers and limited teaching time in the curriculum; and anatomy software is not available in most of the public medical schools because of financial constraint. Moreover, important items that may represent anatomy assessment were excluded from the inventory, although two items in AEEMI-26 measure students’ perception of their confidence in answering anatomy questions. Assessment is typically included as an essential factor of an educational environment. Therefore, future validation may consider conducting a more exhaustive evaluation of the items through item-level refinement. New items that potentially represent the anatomy education environment should be added to the initial pool of items prior to a future cross-validation study. In addition, future validation studies should be conducted in different settings (i.e., different regions and countries).

Inadequacy in the content coverage of the items of the AEEMI-26 may stem from the similarity in the anatomy curricula of the 11 participating medical institutions. In general, these institutions practice integrated curricula, where anatomy is taught in system- or course-based manner with emphasis on horizontal and vertical integration. The teaching methods used to teach anatomy are nearly similar, where the subject is taught through lectures, practical sessions, and problem-based learning. In terms of practical anatomy, nearly all institutions use anatomy models, prosected specimens, and the microscope as teaching tools. Only a few institutions are conducting cadaveric dissections and using anatomy software to supplement teaching. Hence, additional validation studies should be conducted in the future before the AEEMI-26 can be used as a global benchmarking tool. The AEEMI-26 should be validated across countries. Other sources of validity, such as, *consequences* and *relations to other variables* [38], should also be evaluated to ensure the robust psychometric credentials of the AEEMI-26.

## Conclusion

The study illustrated that the AEEMI-26 is a valid and reliable inventory that measures the anatomy education environment. The key strength of this study lies in the involvement of the 1930 medical students who were at different phases of medical training from 11 public and private medical schools in Malaysia. The variation that may exist in the anatomy education environment among the institutions was captured during the validation process, which therefore contributed to the generalizability of the AEEMI-26. Although the study focused on the cross-validation of the AEEMI in the Malaysian context, the AEEMI-26 may be applicable to other countries as is assumes that the anatomy education environment is similar. The findings complement those by Hadie et al. [26], where the inventory was found to have stable constructs. Hence, the AEEMI-26 is useful for enhancing our understanding of the perception of medical students regarding the anatomy education environment. The inventory can be used to obtain students’ feedback on anatomy teaching and learning, and thus serve as a valid benchmarking tool for anatomy education curricula. Further studies should be carried out to validate the AEEMI on a global scale, which will increase its generalizability.

## Supplementary Information

**Additional file 1:.** List of items that were used in this study.

## Data Availability

The datasets used and/or analysed during this study are available from the corresponding author upon reasonable request**.**

## Abbreviations

AEEMI: Anatomy Education Environment Measurement Inventory
CFA: Confirmatory factor analysis RMSEA: Root Mean Square of Error Approximation
CFI: Comparative Fit Index
EFA: Exploratory factor analysis
GFI: Goodness-of-fit Index
HREC: Human Research Ethics Committees
IDP: Institutional development plan
KMO: Kaiser–Meyer–Olkin
MI: Modification indices
NFI: Normed Fit Index
S-CVI/Ave: Scale-level content validity index/average
SPSS: Statistical Package for the Social Sciences
TLI: Tucker–Lewis Index
