# Supplementary material for: Anatomy Education Environment Measurement Inventory (AEEMI): a cross-validation study in Malaysian medical schools
Source: BMC Med Educ. 2021 Jan 14;21:50. doi: 10.1186/s12909-020-02467-w (PMC7807888; doi:10.1186/s12909-020-02467-w)
Supplement: Supplementary file 1 — Additional file 1:. List of items that were used in this study. [file 12909_2020_2467_MOESM1_ESM.docx]

**Additional file 1**

**List of items that were used in this study**

| **Items no** | **Items** |
| --- | --- |
| 1. | The anatomy lectures are interesting. |
| 2. | I am encouraged to participate in anatomy class. |
| 3. | The lectures are simple and easy to understand. |
| 4. | The demonstration session using prosected real specimens are stimulating. |
| 5. | E-learning modules are helpful. |
| 6. | Closed Circuit TV (CCTV) used during demonstration sessions is effective. |
| 7. | Allotted teaching time for anatomy topics is sufficient. |
| 8. | Q/A session at the end of a lecture are helpful. |
| 9. | Presenting anatomy topics to lecturers in student seminars is helpful. |
| 10. | There is lack of interaction during demonstration sessions. |
| 11. | Students Practical Workbooks are not helpful^a^ |
| 12. | The anatomy teaching sessions are teacher-centered. |
| 13. | Anatomy teaching is without clinical application. |
| 14. | Lectures without practical session are sufficient^a^ |
| 15. | Learning objectives for each anatomy topics are clearly spelled out |
| 16. | Learning objectives guide me to learn anatomy. |
| 17. | I used recommended textbooks as main sources for learning anatomy. |
| 18. | I learn anatomy through group discussion. |
| 19. | Learning anatomy prepared me to be a good doctor. |
| 20. | I learn anatomy by drawing anatomical structures. |
| 21. | I use anatomy models/specimens to learn anatomy. |
| 22. | I utilize anatomy museum to learn anatomy. |
| 23. | I learn anatomy by relating the facts to my own body. |
| 24. | I learn anatomy by repetition. |
| 25. | Writing short notes after lectures help in better understanding of the anatomy topics. |
| 26. | I like to understand anatomical structures first before memorizing. |
| 27. | I relied on lecture notes to learn anatomy. |
| 28. | I relied on web-based information to learn anatomy. |
| 29. | Mnemonics are important for memorization of anatomical facts. |
| 30. | Asking questions for clarification is not helpful^a^ |
| 31. | I am confident to answer most of the anatomy questions. |
| 32. | Anatomy questions cover the learning objectives. |
| 33. | Anatomy questions have clinical relevance. |
| 34. | Allocated time to anatomy short essay questions (SEQ) is enough. |
| 35. | Anatomy objective structured practical examination (OSPE) is easy to answer. |
| 36. | Anatomy examinations help me to retain my knowledge for future application use. |
| 37. | Anatomy examinations help me to identify my weaknesses about anatomy knowledge. |
| 38. | Frequency of the examinations is helpful. |
| 39. | End of block assessments strengthen my anatomy knowledge. |
| 40. | Anatomy questions are difficult to understand^a^ |
| 41. | Anatomy questions are difficult^a^ |
| 42. | Short essay questions (SEQ) of anatomy force me to memorize without understanding. |
| 43. | I receive no feedback about my performance in anatomy examinations^a^ |
| 44. | Duration for anatomy objective structured practical examination (OSPE) per station is inadequate^a^ |
| 45. | The content of the anatomy lectures meets the learning objectives |
| 46. | The anatomy topics prepare me for clinical years. |
| 47. | Relevant anatomy topics are reemphasized in clinical years. |
| 48. | The anatomy topics are relevant to future profession. |
| 49. | The facts presented in the anatomy lectures are contradictory to the recommended textbooks^a^ |
| 50. | Anatomy content in the lectures is superficial. |
| 51. | Too many topics to be learnt within limited time. |
| 52. | Anatomy topics with less clinical application are given more emphasis. |
| 53. | The teachers simplify difficult anatomy concepts. |
| 54. | Teachers are approachable. |
| 55. | Teachers are knowledgeable. |
| 56. | Teachers are well prepared. |
| 57. | Teachers know how to make session interesting. |
| 58. | Teachers are enthusiastic to teach. |
| 59. | Teachers inspire me to learn more. |
| 60. | Teachers speak clearly. |
| 61. | Teachers are creative in using various teaching tools to stimulate learning. |
| 62. | Teachers stimulate confidence. |
| 63. | Teachers are good role model for learning anatomy. |
| 64. | Anatomy teachers speak in monotonous voice while delivering lecture. |
| 65. | Teachers get irritated when asked questions^a^ |
| 66. | Teachers scold for mistakes^a^ |
| 67. | Teachers avoid eye contact. |
| 68. | Teachers are friendly. |
| 69. | Lecture hall is comfortable. |
| 70. | Anatomy museum is accessible. |
| 71. | Learning facilities are well maintained. |
| 72. | Anatomy teachers are available to help students. |
| 73. | My friends help me to improve my understanding on anatomy subjects. |
| 74. | Anatomy museum is conducive for learning. |
| 75. | There are too many students per group during practical session. |
| 76. | Practical sessions are well organized. |
| 77. | Supporting staff are uncooperative^a^ |
| 78. | The teachers criticize students when they make errors. |
| 79. | Absenteeism in anatomy teaching sessions is common^a^ |
| 80. | Practical sessions are too long^a^ |
| 81. | The atmosphere in anatomy teaching sessions is stressful. |
| 82. | Practical laboratory/dissection hall is conducive for learning anatomy |
| 83. | The plastic models provide me with good understanding of the gross anatomy structures^a^ |
| 84. | I have to compete for anatomy models during practical session. |
| 85. | Learning histology slides using computer software is useful. |
| 86. | The prosected specimens are adequate. |
| 87. | The prosected specimens are accessible. |
| 88. | The anatomy models reflect the real structures. |
| 89. | Plastinated specimens are helpful in understanding the relationship of various structures. |
| 90. | Fixed Learning Module (FLM) is effective in understanding anatomical structures^a^ |
| 91. | Anatomy plastic models are adequate in number^a^ |
| 92. | Bone sets provided are useful in understanding the gross anatomical structures. |
| 93. | Quality of the microscopes provided to study histology slides is poor. |
| 94. | Inadequate number of histology slides. |
| 95. | Poor quality of histology slides. |
| 96. | Anatomy plastic models are not well maintained^a^ |
| 97. | Computer aided learning tools (e.g 3D Anatomy and Virtual Dissection) are not available. |
| 98. | Cadaver dissection sessions are not available. |
| 99. | Anatomy is an interesting subject. |
| 100. | Learning anatomy is fun. |
| 101. | I look forward to attend anatomy classes. |
| 102. | I spend a lot of time in learning anatomy. |
| 103. | Anatomy subject gives me feeling of becoming a doctor. |
| 104. | I like to ask questions during anatomy classes. |
| 105. | I feel bored during anatomy classes^a^ |
| 106. | I learn anatomy just to pass examinations^a^ |
| 107. | I can explain difficult anatomy concepts to my friends |
| 108. | I am confident to teach anatomy to others. |
| 109. | I am confident to answer anatomy questions well. |
| 110. | I have difficulty to understand most of the anatomy topics |
| 111. | It is beyond my capability to understand anatomy^a^ |
| 112. | I need extra guidance to learn anatomy. |
| 113. | I need extra effort to understand anatomy subject |
| 114. | My anatomy knowledge helps me to understand other medical subjects. |
| 115. | I can apply my anatomical knowledge in clinical years. |
| 116. | My histology knowledge makes me understand the histopathology. |
| 117. | I rely on gross anatomy knowledge to learn embryology. |
| 118. | I apply my anatomy knowledge while conducting physical examination on patients. |
| 119. | I apply my anatomy knowledge while performing clinical procedures. |
| 120. | I cannot relate the anatomy knowledge to the clinical problems during the PBL discussion^a^ |
| 121. | I do not rely on gross anatomy knowledge to learn histology^a^ |
| 122. | I spend reasonable time to revise anatomy topics. |
| 123. | I know how to revise anatomy topics^a^ |
| 124. | I rely on group discussion for revision. |
| 125. | The revision class helped me to understand the subjects I found most difficult. |
| 126. | I am given sufficient time for revision prior to examination. |
| 127. | I use past-years questions to prepare for examinations. |
| 128. | I revise anatomy topics late before examination^a^ |
| 129. | I give less priority to anatomy revision^a^ |
| 130. | Teachers are helpful |
| 131. | I learn anatomy through reflection |
| 132. | Anatomy is taught in clinical context |
